# Dependency of the drag coefficient on boundary layer stability beneath drifting sea ice in the central Arctic Ocean

**DOI:** 10.1038/s41598-024-66124-8

**Published:** 2024-07-04

**Authors:** Yusuke Kawaguchi, Mario Hoppmann, Kunio Shirasawa, Benjamin Rabe, Ivan Kuznetsov

**Affiliations:** 1 Atmosphere and Ocean Research Institute, The University of Tokyo, 5-1-5 Kashiwa-no-ha, Chiba, 277-8564 Japan; 2 Alfred-Wegener-Institut Helmholtz-Zentrum für Polar- und Meeresforschung, Bremerhaven, Germany; 3 Institute of Low Temperature Science, Hokkaido University, Sapporo, Japan

**Keywords:** Ice-ocean boundary layer, Drag coefficient, Turning angle, Static stability, Heat balance, Sea ice dynamics, Arctic Ocean, Physical oceanography, Climate and Earth system modelling, Cryospheric science

## Abstract

The ice-ocean drag coefficient $C_{w}$ and turning angle $\theta_{w}$ are crucial parameters in ice-ocean coupled simulations, determining the transfer of momentum between the two media. These parameters are often treated as constants regardless of the static stability at the ice-ocean interface. This study investigates the variability of $C_{w}$ and $\theta_{w}$ based on direct observations of thermal and kinetic energy balance. The observations were conducted beneath multiyear ice packs widely across the central Arctic during a period transitioning from ablation to refreezing, indicating significant variability of $C_{w}$ = 1–130 $\times$ 10^−3^ and $\theta_{w}$ =  − 19–1° at 5 m depth. Comparing different stations, the observations suggest a pronounced dependence of $C_{w}$ on the stability parameter ($\mu$) resulting from mechanical and buoyant forcing. $C_{w}$ rapidly decays with increasing $\mu$, indicating that the ice-to-ocean momentum transfer is enhanced for neutral or unstable conditions, while it is weakened for stable conditions. In addition, observed vertical profiles of currents revealed that $|\theta_{w}|$ tends to be smaller for unstable and larger for stable conditions. We suggest that numerical simulations using constant values could result in an underestimate of large-scale near-surface currents during the ice growing period.

## Introduction

During recent decades, the sea ice extent in the Arctic Ocean has been diminishing faster than predicted by the forecasts from global climate model simulations e.g.,^1–5^. In the ice-ocean boundary layer (IOBL), which lies under drifting sea ice, the Coriolis force causes the flow to turn due to the internal friction. Therefore, it requires the determination of the corresponding parameters of the drag coefficient ($C_{w}$) and the turning angle ($\theta_{w}$)^6–10^. With those parameters, the momentum transfer, known as the Reynold stress ($\tau_{w}$), is typically formulated by the differential motion between sea ice and near-surface water:

1 $$\tau_{w} = C_{w} \left( {U_{i} - U_{w} } \right)\left| {U_{i} - U_{w} } \right|{\text{exp}}\left( { - i\theta_{w} } \right),$$

where $U_{i}$ is the ice drift, and $U_{w}$ is the ocean current underneath the drifting sea ice in the complex form; $\theta_{w}$ is positive for counterclockwise rotation. The near-surface Ekman current rotates clockwise in the Northern Hemisphere ($\theta_{w} < 0$) and vice versa in the Southern Hemisphere ($\theta_{w} > 0$). For neutral stratification, $\left| {\theta_{w} } \right|$ reportedly ranges within 15–25°^11^.

In the ice-ocean coupled simulations, $C_{w}$ and $\theta_{w}$ are the most important parameters that influence the exchange of momentum and kinetic energy between the two media, ultimately affecting the large-scale circulation of fresh water and ice export towards the mid-latitude seas^11–19^. The exchange rate of momentum, represented by $C_{w}$, should be inherently variable in association with the state of static stability, which depends on the melting or refreezing of sea ice at its bottom^20,21^. However, many numerical models used to predict the pan-Arctic climate assume it to be constant, regardless of time and location^22,23^. This knowledge gap is mainly rooted in the lack of direct observations of the spatiotemporal variations in $\tau_{w}$ and $C_{w}$. Hence, there is a critical need to reevaluate those parameters in the context of the ongoing global warming and diminishing sea ice extent in the Arctic Ocean^17,18^.

For the bulk estimate of interfacial turbulent fluxes, particularly when using data from autonomous drifting buoys, the differential velocity between the ice and ocean is commonly neglected^24–27^. In such instances, the stress is frequently parameterized solely by the sea ice drift ($U_{i}$) with some empirical constants, e.g., $\tau_{w} = 0.0104U_{i}^{1.78}$^24,25^. Alternatively, it can be determined by solving the implicit equation of Rossby similarity assuming a neutral IOBL^27^: $\frac{{U_{i} }}{{u_{0}^{*} }} \propto ln \left( {\frac{{u_{0}^{*} }}{{fz_{0} }}} \right)$, where $u_{0}^{*} = \sqrt {\tau_{w} }$ is the interfacial friction velocity, $f$ is the Coriolis frequency, and $z_{0}$ is the surface roughness.

In the Arctic Ocean, there are relatively few reports of the drag coefficient based on direct measurements of the Reynolds stress e.g.,^11,12,28–32^. For example, Shirasawa and Ingram^30^ proposed a value of $C_{w}$ = 5 × 10^−3^ at 1 m beneath the ice bottom in the Barrow Strait of the Canadian Arctic. Some numerical models solve the ‘law of the wall’ (*LoW*) problem for the determination of $C_{w}$ assuming a logarithmic current profile in the vertical direction under a neutrally stratified IOBL, i.e., $\sqrt {C_{w} } = \left( {\frac{1}{\kappa }ln \left| {\frac{z}{{z_{0} }}} \right| } \right)^{ - 1}$, where $\kappa$ is the von Kármán constant, and $z$ is the vertical coordinate and points upward. For $z$ =  − 2.0 m, the above model yields an estimate of $C_{w}$ = 12 $\times$ 10^−3^^27^. Cole et al.^12^ performed year-long measurements of $C_{w}$ and $\theta_{w}$ utilizing autonomous drifting buoys in the marginal ice zone of the Canada Basin. They found seasonal and regional variations in these parameters, particularly noting an anomalously reduced $C_{w}$ during the melting season compared to the rest of the year. Heorton et al.^11^ evaluate those parameters in inverse modelling techniques based on the momentum balance of freely drifting sea ice, proposing an average value of $C_{w}$ = 2.4 × 10^−3^.

From the viewpoint of the steady-state momentum balance of the sea ice, the drag coefficients may significantly affect the ice drift, as described by the relation below^9,33^:

2 $$U_{i} = NaU_{a} {\text{exp}}\left( { - i\left( {\theta_{w} - \theta_{a} } \right)} \right) + U_{wg} ,$$

where $Na = \sqrt {\rho_{a} C_{a} /\rho_{w} C_{w} }$ is the Nansen number; $U_{a}$ is the surface wind, $C_{a}$ is the air-ice drag coefficient; $\rho_{a}$ are $\rho_{w}$ are the densities of air and water, $U_{wg}$ is the geostrophic current, and $\theta_{a}$ is the turning angle in air. We assume that the ice internals tress is ignorable compared to the other kinematic terms, resulting in the state of free drift^9^. In Eq. (2), $Na$ can be regarded as the wind factor representing the ice drift relative to the surface wind speed^34–36^, assuming a negligible geostrophic current, i.e., $U_{wg} \approx$ 0. If the stability state differs between the fluids below and above the ice layer, the ice drift speed could vary significantly through $Na$.

**Figure 1:**
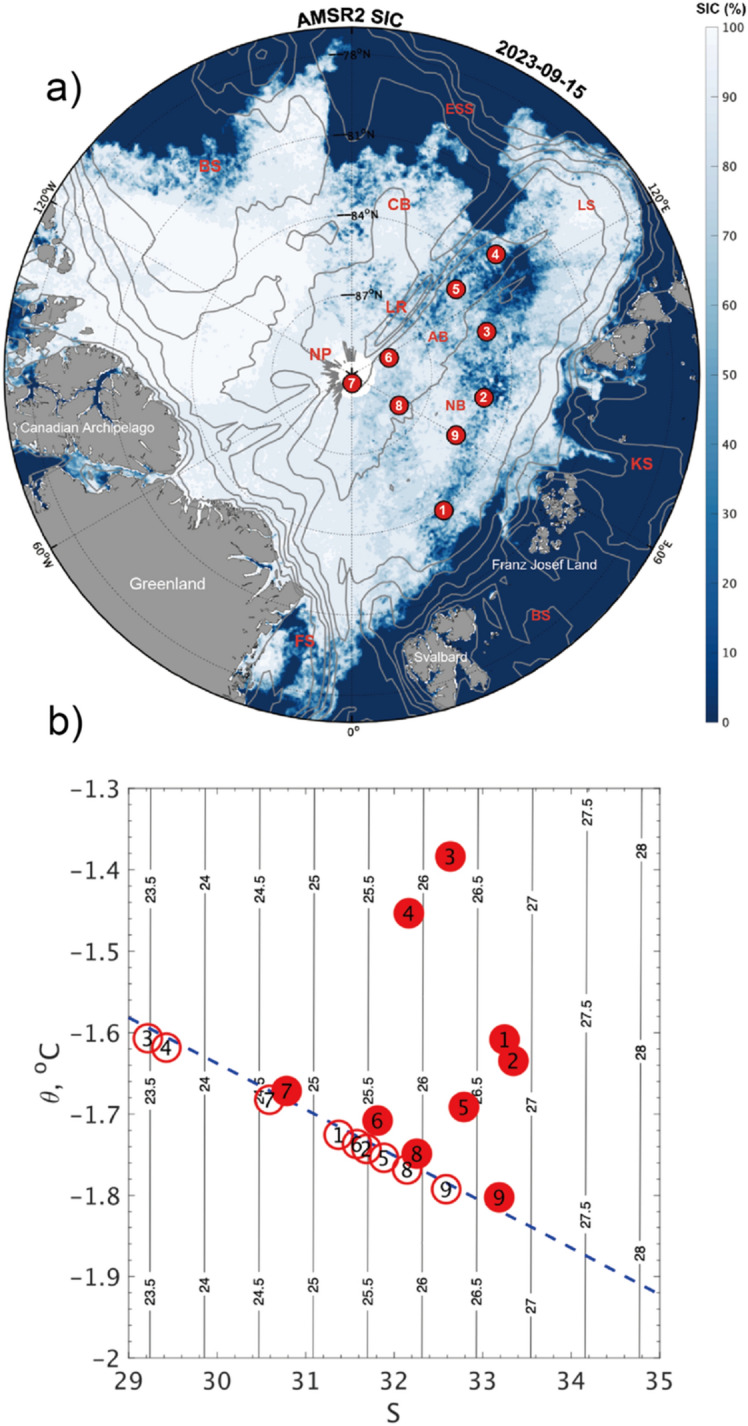
(**a**) Map of the study area, including sea ice concentration on September 15, 2023. Red dots represent Stations #1–9. Gray contours show the IBCAO bottom relief:100, 500, 1000 and 2000 m. (**b**) Potential temperature and salinity of sea water at each station, at depths within mixed layer (filled circles; $T_{w}$ and $S_{w}$, respectively) and the ice-ocean interface (blank circles; $T_{0}$ and $S_{0}$, respectively). Contours show the potential density anomaly.

This study aims to derive the horizontal distribution of $C_{w}$ and $\theta_{w}$ across roughly 2500 km in the central Arctic Ocean using measurements obtained during the RV *Polarstern* expedition PS138 in 2023 (Fig. 1);^37,38^. We explore the relationship of the key variables $C_{w}$ and $\theta_{w}$ against the static boundary condition at the ice-ocean interface and address the question of how the static stability parameter related to basal melting and refreezing can affect the exchange of momentum across the ice-ocean interface and ultimately influence the large-scale circulation in the central Arctic basins.

## Results

We conducted under-ice observations at nine ice stations during the PS138 cruise in the Eurasian Basin, encompassing the Amundsen and Nansen Basins (see Methods for further details; Fig. 1a). The observations were based on the eddy-covariance technique, which directly measured the three-dimensional movement of seawater in a small sampling volume beneath drifting ice floes. Based on these data, we calculated the cross-spectral power for the turbulent fluxes of kinematic and thermal energy, respectively represented by $\tau_{w} = \langle u^{\prime}w^{\prime}\rangle^{2} + \langle v^{\prime}w^{\prime}\rangle^{2}$ and $F_{h} = \rho_{w} C_{p} \langle w^{\prime}T^{\prime}\rangle$. In the following sections, we will display the results of $C_{w}$, which is calculated from the relation in Eq. (1), based on the ECS measurement of $\tau_{w}$ and the ice-referenced velocity $U_{0}$. Furthermore, the turning angle ($\theta_{w}$) is estimated from vertical profiles of horizontal current based on an ice-fixed acoustic Doppler current profiler (ADCP) (see the corresponding subsection in the Methods).

### Turbulent fluxes from ECS observations

**Figure 2:**
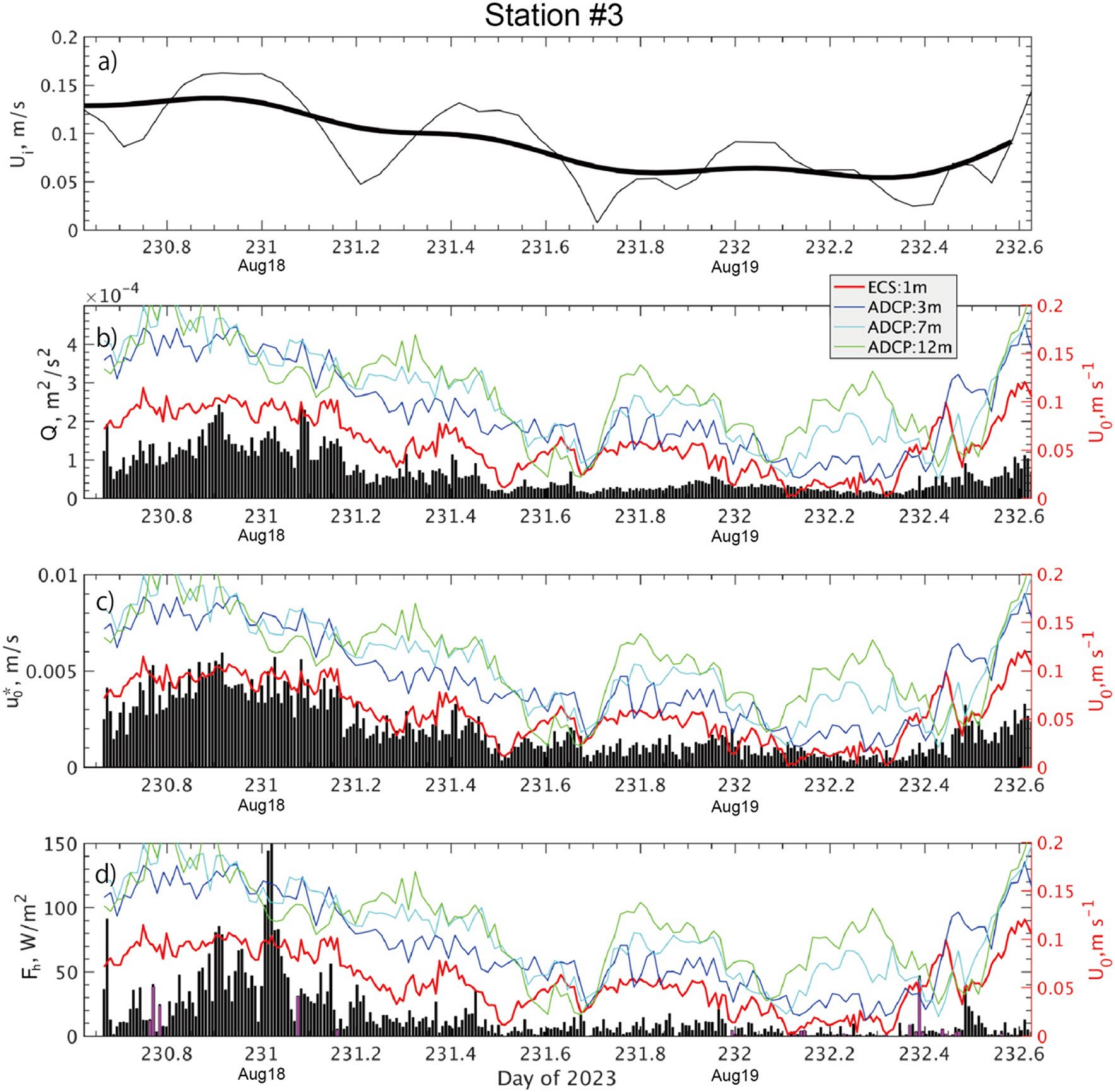
Time series of turbulent fluxes from the ECS observations at Station #3: (**a**) ice drift intensity (thin line: unfiltered; bold line: 12-h lowpass filter); (**b**) turbulent kinetic energy ($Q$); (**c**) friction velocity ($u_{0}^{*}$); (**d**) turbulent heat flux ($F_{h}$). In panels (b-d), red lines show mean current intensity ($U_{0}$) directly measured by ECS, while colored lines represent ADCP measurements at levels of 3, 7 and 12 m (see legend for corresponding depths). In (**d**), magenta vertical bars denote negative $F_{h}$.

For Stations #1 to #5, all turbulence-related variables obtained by the eddy-covariance system (ECS) are well correlated with the mean current intensity $U_{0}$ (equivalent to $U_{w} - U_{i}$) directly measured from the drifting sea ice platform (Fig. 2; Figs. S1–S9). These include turbulent kinetic energy $\left( {Q = \frac{1}{2}\left( {u^{\prime 2} + v^{\prime 2} + w^{\prime 2} } \right)} \right)$, interfacial friction velocity ($u_{0}^{*} = \sqrt {\tau_{w} }$), and turbulent heat flux ($F_{h}$), where ($u^{\prime } , v^{\prime } , w^{\prime }$) is the eddy component of the ocean current observed in the ice-fixed frame, $T^{\prime}$ is the eddy component of the seawater temperature, $\rho_{w}$ the seawater density, and $C_{p}$ the specific heat. For example, in the case of Station #3, the time series of burst observation confirms a clear concurrency between $U_{0}$ and the low-passed ice drift ($U_{i}$), which were measured by independent instrumentations (Fig. 2a,b). Furthermore, $Q$ and $u_{0}^{*}$ exhibit corresponding temporal variations with $U_{0}$ and consequently $U_{i}$ (Fig. 2b,c). In other words, the faster the ice floe drifted, the more energetic the turbulent mixing became. At Station #3, the variation in $F_{h}$ is well explained by $U_{i}$, rather than by the change in local temperature immediately underneath the ice bottom (Figure not shown).

**Figure 3:**
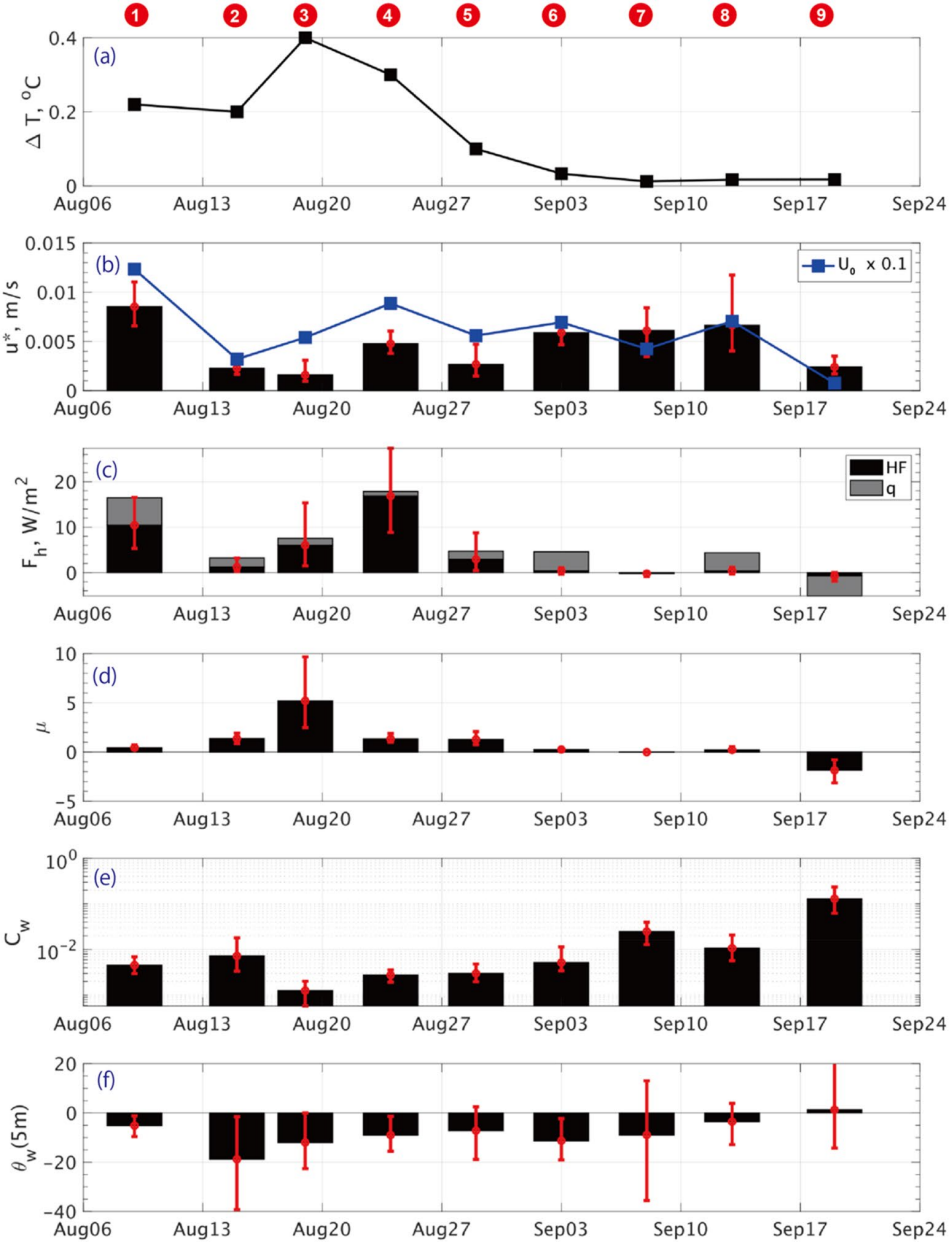
Overview of statistics for kinematic and thermodynamic parameters during all stations: (**a**) temperature deviation from the freezing point ($\Delta T = T_{w} - T_{f}$), (**b**) friction velocity ($u_{0}^{*}$), (**c**) turbulent heat flux ($F_{h} = \rho C_{p} \langle w'T'\rangle$) in black and conductive heat flux ($\dot{q}$) in gray, (**d**) stability parameter (${\upmu } = L_{PB} /L_{O}$), (e) drag coefficient ($C_{w}$) and (**f**) turning angle ($\theta_{w}$) at 5 m depth. Bold black bars indicate medians with red error bars representing first-to-third quartiles. Numbers in red circles at the top denote the corresponding station. In (**b**), blue line shows mean current intensity, $U_{0}$, divided by 10.

The friction velocity ($u_{0}^{*}$) varied moderately from station to station along with $U_{0}$, as shown in Fig. 3b. Meanwhile, $F_{h}$ exhibited pronounced changes in time and location. Results from the earlier stations, particularly Stations #1, 3, and 5, reveal that a substantial amount of heat was transferred from the ocean to the ice bottom through turbulent mixing. In particular, the results from Station #4 show a median estimate of $F_{h}$ = 15 W m^−2^ with an interquartile range of 30 W m^−2^, when the floe was in a region with a discernibly reduced ice concentration (Fig. 1a). During those stations, the far-field water temperature ($T_{w}$) was above the freezing point ($T_{f}$), determined by the practical salinity ($S_{w}$) at the same depth, where the gap between $T_{w}$ and $T_{f}$ fell within the range between 0.2 and 0.4 K (Fig. 3a).

In contrast to the earlier stations, Stations #6 to #9 exhibited extremely small or nearly zero $F_{h}$ (Fig. 3c). This can be explained by the presence of near-freezing seawater in the upper part of mixed layer ($T_{w} \approx T_{f} )$ (Fig. 3a). Especially for Station #9, the observed $F_{h}$ was mostly negative, where turbulent heat flux was directed downward at the interface, i.e., from the ice to the ocean.

The ECS observations exhibited that the medians of the drag coefficient $C_{w}$ largely increased with increasing station number, from the value of $C_{w} =$ 1 $\times$ 10^−3^ to 130 $\times$ 10^−3^, transitioning from early August to late September (Fig. 3e). Meanwhile, the turning angle $\theta_{w}$ generally diminished in terms of its magnitude, e.g., from $\theta_{w} =$ − 19 $^\circ$ to 1 $^\circ$ at 5 m depth, with the exception of the relatively small deviation of $\theta_{w} =$  − 5 $^\circ$ at Station #1 (Fig. 3f).

### Conductive and latent heat

**Figure 4:**
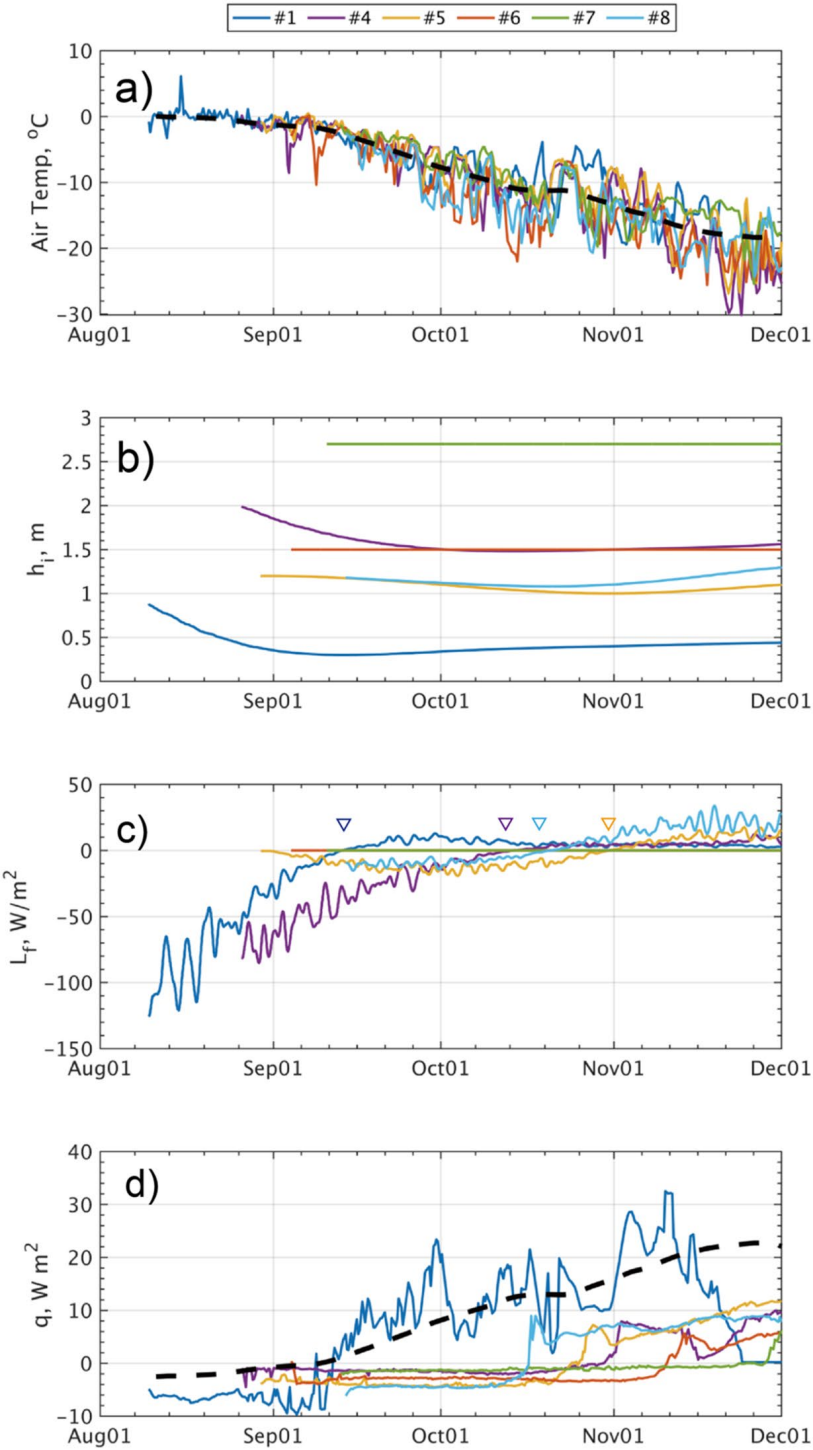
Time series of SIMBA-derived variables: (**a**) air temperature $T_{a}$, (**b**) ice thickness $h_{i}$ (**c**) latent heat $L_{f}$ and (**d**) conductive heat flux $\dot{q}$. In (**a**), a black dashed curve denotes an average of $T_{a}$ from the six SIMBA buoys, while in (**d**) it denotes $\dot{q}$ derived from the averaged $T_{a}$ and the constant ice thickness of 1.2 m. In (**c**), inverted triangles denote zero-crossing points in $L_{f}$.

The SIMBA ice mass balance buoys (refer to Methods) demonstrated the temporal variation of ice thickness ($h_{i}$) as the ice floes drifted (Fig. 4b). For example, Station #1 exhibited $h_{i}$ decreasing by more than 0.4 m for three weeks in August until it underwent a transition to the growing phase around 10th in September (inverted triangles in Fig. 4c). Stations #4 and #8 switched to the growth in mid-October, while Station #5 did that in late October. At the remaining stations of #6 and #7, $h_{i}$ remained relatively constant through the end of the year.

According to the time evolution in $h_{i}$, detected by tracking the SIMBA’s ice bottom, the latent heat ($L_{f}$) can be estimated (refer to Methods). For Stations #1 and #4, $L_{f}$ exceeded as large as more than 50 W m^−2^ in negative values during the melting period (Fig. 4c). For Stations #6 and #7, deployed in the highest latitudes, $L_{f}$ is estimated to be nearly zero through the year. The rest of stations (#5 and #8) showed reduced variability in $L_{f}$ being less than 10 W m^−2^ in magnitude.

From the vertical gradient of ice temperatures $\left( {T_{i} \left( z \right)} \right)$ (Fig. S10), before the mid-September, the conductive heat flux ($\dot{q}$) was directed downward at most stations, typically ranging from $\dot{q}$ =  − 4 to 0 W m^−2^ (Fig. 4d). Particularly, Station #1 exhibited an anomalously significant downward flux, exceeding $\dot{q}$ $\approx$ − 6 W m^−2^ (Fig. 4b).

At Stations #2, 3 and 9, there are no records of $T_{i}$ available for the calculation in terms of $\dot{q}$. We hence estimated $\dot{q}$ by assuming a linear vertical profile of $T_{i}$, between the measured near-surface air temperature ($T_{a}$) and the freezing-cold temperature in water, i.e., $T_{w} \sim T_{f}$ (Fig. 4a) (see Methods). Using this approximation, we derived the rough estimates of $\dot{q} =$ − 2.0, − 1.5 and 4.4 W m^−2^, respectively for Stations #2, 3 and 9 (Table 1).

**Table 1:** Overview of under-ice observations during the *Polarstern* ArcWatch expedition, 2023. Turning angles ($\theta_{w}$) are shown for depths of 5 m and 10 m in the top and bottom rows, respectively. Numbers inside and outside parentheses denote medians and interquartile ranges, respectively.

Station #	1	2	3	4	5	6	7	8	9
Latitude [$^\circ$ N]	84.07	84.95	84.76	82.90	85.05	88.49	89.94	87.93	85.47
Longitude [$^\circ$ E]	31.21	80.14	107.79	129.99	130.35	111.93	–15.22	60.32	59.97
Duration [h]	39.8	29.3	48.2	23.2	63.3	24.5	49.8	25.7	19.0
$u_{0}^{*} \times$ 10^−4^ [m s^−1^]	85 (45)	23 (14)	16 (23)	48 (23)	27 (32)	59 (21)	61 (50)	66 (77)	24 (18)
$F_{h}$ [W m^−2^]	10.5 (11.2)	1.2 (2.9)	6.0 (13.9)	16.9 (18.5)	2.9 (8.3)	0.4 (1.4)	− 0.2 (1.0)	0.4 (1.5)	− 0.7 (1.9)
$\dot{q}$ [W m^−2^]	− 6.0	− 2.0	− 1.5	− 1.0	− 1.8	− 4.2	0.0	− 4.0	4.4
$\langle w^{\prime}b^{\prime}\rangle \times$ 10^−9^ [m^2^ s^−3^]	11.0 (7.2)	2.2 (1.9)	4.8 (8.6)	11.1 (11.5)	3.1 (5.3)	3.0 (0.9)	− 0.1 (0.6)	2.9 (0.9)	− 3.5 (1.2)
$\mu$	0.4 (0.5)	1.4 (1.1)	5.2 (7.2)	1.3 (0.9)	1.3 (1.3)	0.3 (0.2)	0.0 (0.1)	0.2 (0.5)	− 1.9 (2.4)
$C_{w}$ $\times$ 10^−3^	4.6 (4.0)	7.3 (14.7)	1.3 (1.5)	2.8 (1.7)	3.0 (2.9)	5.2 (0.8)	25.0 (26.7)	10.7 (15.1)	130.3 (173.0)
$\theta_{w}$ [$^\circ$]	− 5.2 − 11.2	− 18.8 − 16.5	− 12.0 − 15.5	− 9.0 − 15.9	− 7.2 − 11.0	− 11.3 − 26.0	− 9.0 − 6.4	− 3.5 − 8.0	1.3 7.8

For Stations #1 and #4, the measured $F_{h}$ values were so small (10 and 17 W m^−2^, respectively) that they cannot explain the rapid decay of the ice column at the undersurface (Table 1). The underrated $F_{h}$ based on the ECS would be attributed to some environmental factors. Oceanic heat content stored in the fresh meltwater, which lies between the ice and the ECS instruments, has the potential to facilitate basal ablation^39^.

### Static stability near the interface

We now address the static stability issue drawing from the measured heat and momentum balance. To characterize the stability state in IOBL, the introduction of the Obukhov length scale as

3 $$Lo = u_{0}^{*3} /\left( {\kappa \langle w^{\prime } b^{\prime } \rangle } \right)$$

is advantageous, where $\langle w'b'\rangle$ represents the turbulent buoyancy flux at the interface. The buoyancy flux ($\langle w'b'\rangle$) is estimated based on thermohaline conservation at the interface, utilizing the hydrographic observations for the far-field temperature and salinity (refer to Methods) as well as the ice mass balance observations for the conductive heat flux.

In general, $\langle w^{\prime } b^{\prime } \rangle$ concurrently varied with $F_{h}$ (Table 1). For the early stations (Stations #1 to #5), which occurred in August, the ice-ocean interface exhibited $\langle w^{\prime } b^{\prime } \rangle > 0$, likely due to the significant melting of sea ice at its bottom, resulting in the stabilized boundary condition. Conversely, for the later stations (#6 to #9), $\langle w^{\prime } b^{\prime } \rangle$ was estimated to be significantly lowered. Especially, for Stations #7 and #9, $\langle w^{\prime } b^{\prime } \rangle$ indicated slightly negative values. Thus, the interfacial boundary condition can be perceived as transitioning from stable to neutral and/or even slightly unstable.

We then transform $Lo$ into a non-dimensional form, known as the stability parameter ($\mu$)^27^ (Fig. 3d):

4 $$\mu = \frac{{L_{PB} }}{{L_{O} }} = \frac{{\kappa \langle w^{\prime } b^{\prime } \rangle }}{{fu_{0}^{*2} }},$$

where $\mu$ represents the ratio of the planetary boundary layer thickness $\left( {L_{PB} = \frac{{u_{0}^{*} }}{f}} \right)$ to $L_{O}$. Equation (4) is formulated to draw an analogy with the physics of the air-ice boundary layer^40,41^. General interpretation of physics can describe that $\mu > 0$ indicates a stable boundary condition, typically associated with ice ablation, while $\mu < 0$ indicates an unstable condition with brine rejection due to ice growth. Hence, $\mu = 0$ signifies a neutral boundary flux condition. Using the observed $u_{0}^{*}$ (Table 1), we also note that the mean value of $L_{PB}$ can vary between 110 and 580 m. According to the observations, the earlier group (#1–5) in August typically exhibited positive values of $\mu$ ranging between the orders of 0.1 and 1, indicating a stable boundary condition. In the meantime, the latter group (#6–9) in September showed nearly zero or slightly negative values of $\mu$, respectively indicating neutral or statically unstable boundary conditions (Fig. 3d).

### Vertical structures of Ekman currents

**Figure 5:**
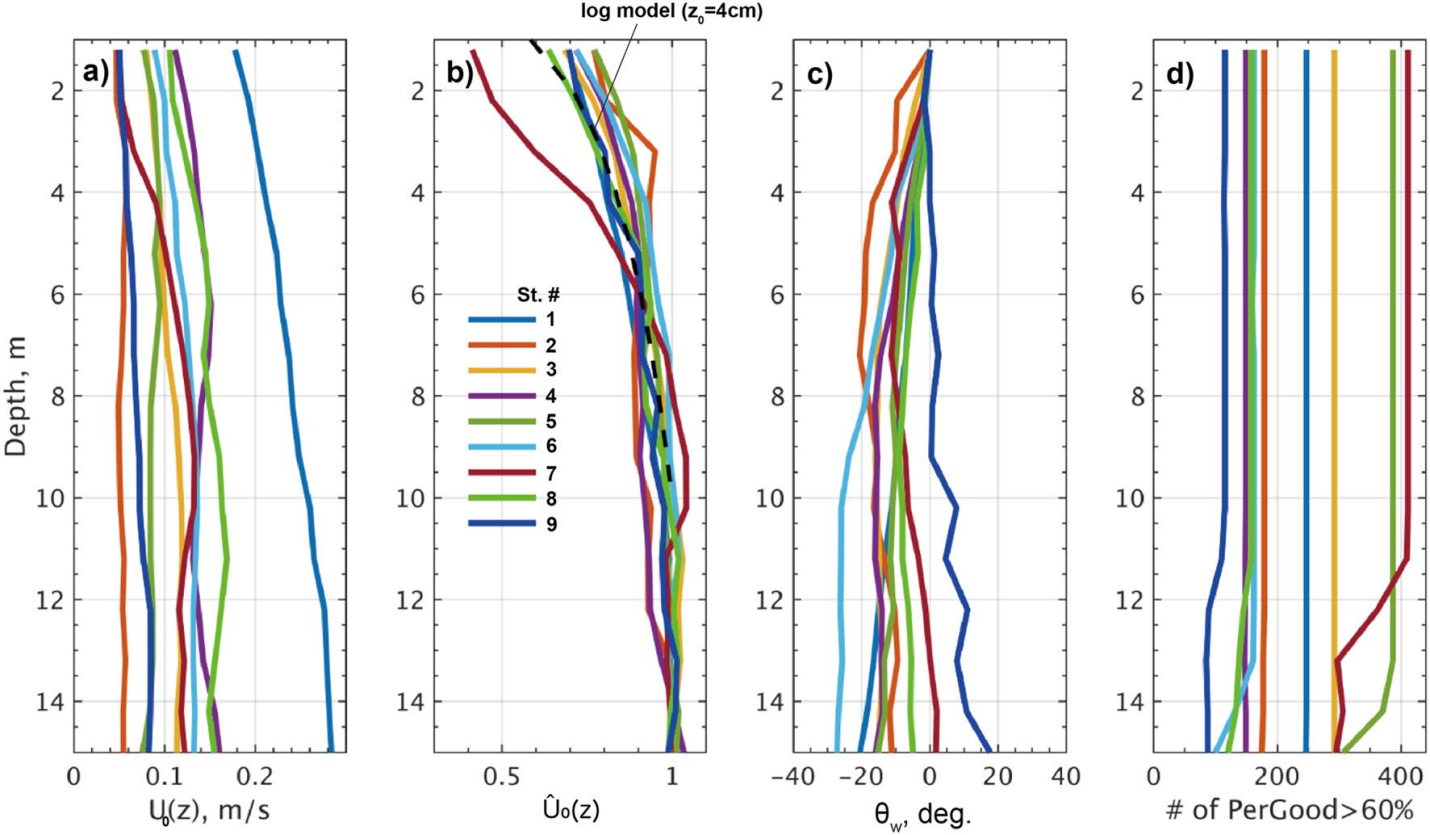
Vertical profiles of medians of horizontal current velocity from Stations #1 to #9: (**a**) current magnitude $U_{0} \left( z \right)$, (**b**) its normalized deviation ($\hat{U}_{0} \left( z \right) = \left( {U_{0} - U_{geo} } \right)/U_{geo}$), where $U_{geo}$ is the geostrophic current averaged over depths of 15–20 m, and (**c**) turning angle $\theta_{w}$ relative to the near-surface current, where positive values indicate counterclockwise rotation, and (**d**) number of quality data where Percent Good ≥ 60%. In (**b**), a black dashed curve represents the *LoW* logarithmic model, with a surface roughness of $z_{0}$ = 4 cm.

The vertical structure of horizontal currents in IOBL was examined using data from an ADCP (Fig. 5). The measured current speed ($U_{0}$) relative to the drifting sea ice exhibited a characteristic vertical variation of the *LoW* theory, logarithmically increasing with depth^27^:

5 $$\hat{U}_{0} \left( z \right) = \frac{{u_{0}^{*} }}{\kappa }ln \left( {\left| {\frac{z}{{z_{0} }}} \right|} \right){\text{exp}}\left( { - i\theta_{w} } \right)$$

The current strength increased with depth and reached an equilibrium level around a depth of 10 m, where the geostrophic current ($U_{geo}$) was expectedly dominant (Fig. 5a). For the normalized vertical profile of ${\hat{U}_{0} =( U_{0} \left( z \right) - U_{geo} } ) /U_{geo}$, where $U_{geo}$ is derived as an average of $U_{0} \left( z \right)$ over a depth of 15 to 20 m, the surface roughness of $\left| {z_{0} } \right|$= 4 cm^27^ for the logarithmic model above is in good agreement with the observed vertical profile (Fig. 5b).

Comparing current speeds at different levels as detected by the ADCP (colored curves in Fig. 2b–d), it is evident that the ECS instrument was positioned in the middle of the *LoW* boundary layer, where the ice-relative current strength logarithmically increases along the depth (Fig. 5b). The current observed at the ECS level, typically 0.7 m below the ice bottom, was as small as 0.3 to 0.6 times the current at 12 m depth.

In the present study, the ADCP observations reveal that the horizontal current vector was rotated clockwise relative to the surface current and mostly increased linearly with depth, as expected from the classical Ekman theory^5^ (Fig. 5c). From Stations #1 to #8, the observation show rotation angles ranging from $\theta_{w} =$  − 18.8° to − 3.5° and from $\theta_{w} =$  − 26° to − 6.4°, respectively at 5 and 10 m depth (Fig. 3f). We note that $\theta_{w}$ is a deviation from horizontal current at the near-surface level observed by the ice-fixed ADCP. This finding is generally consistent with previous reports, such as $\left| {\theta_{w} } \right| =$ 17° in the Baltic Sea^22^ and $\left| {\theta_{w} } \right| =$ 15° in the Weddell Sea^19^.

## Discussions

### Dependency of $C_{w}$ and $\theta_{w}$ on stability parameters

**Figure 6:**
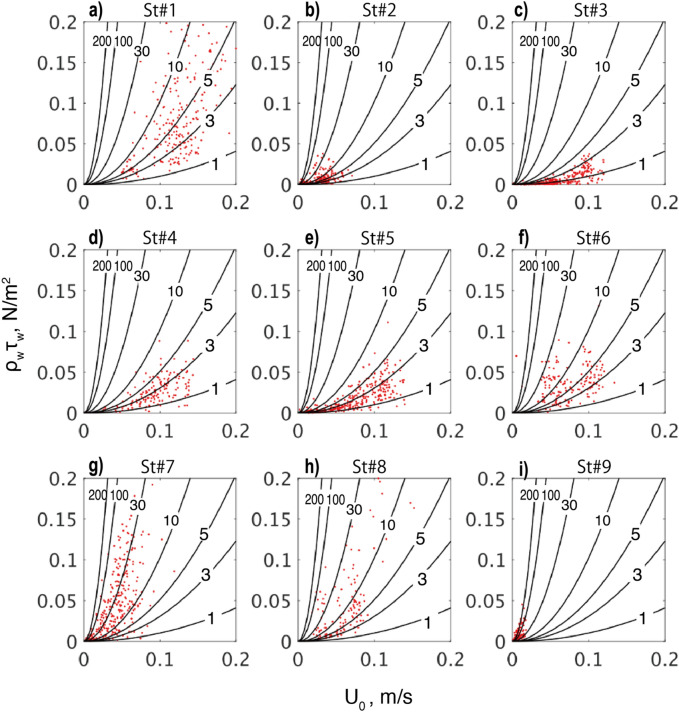
Relationship between the current speed ($U_{0}$) measured from the ice-fixed frame and the ice-ocean Reynolds stress ($\rho_{w} \tau_{w}$) for each ice station. Black curves show the drag coefficients ($C_{w} \times$ 10^3^) for the quadratic model (Eq. 1).

Based on Eq. (1), the relationship between the ice-referenced current ($U_{0}$) and the Reynolds stress ($\tau_{w}$) is investigated for all ice stations (Fig. 6). For every ice floe, there was a clearly positive relation between the two variables, where the stress $\tau_{w}$ displays a quadratic growth with the mean relative current $U_{0}$ (Fig. 6a-i).

**Figure 7:**
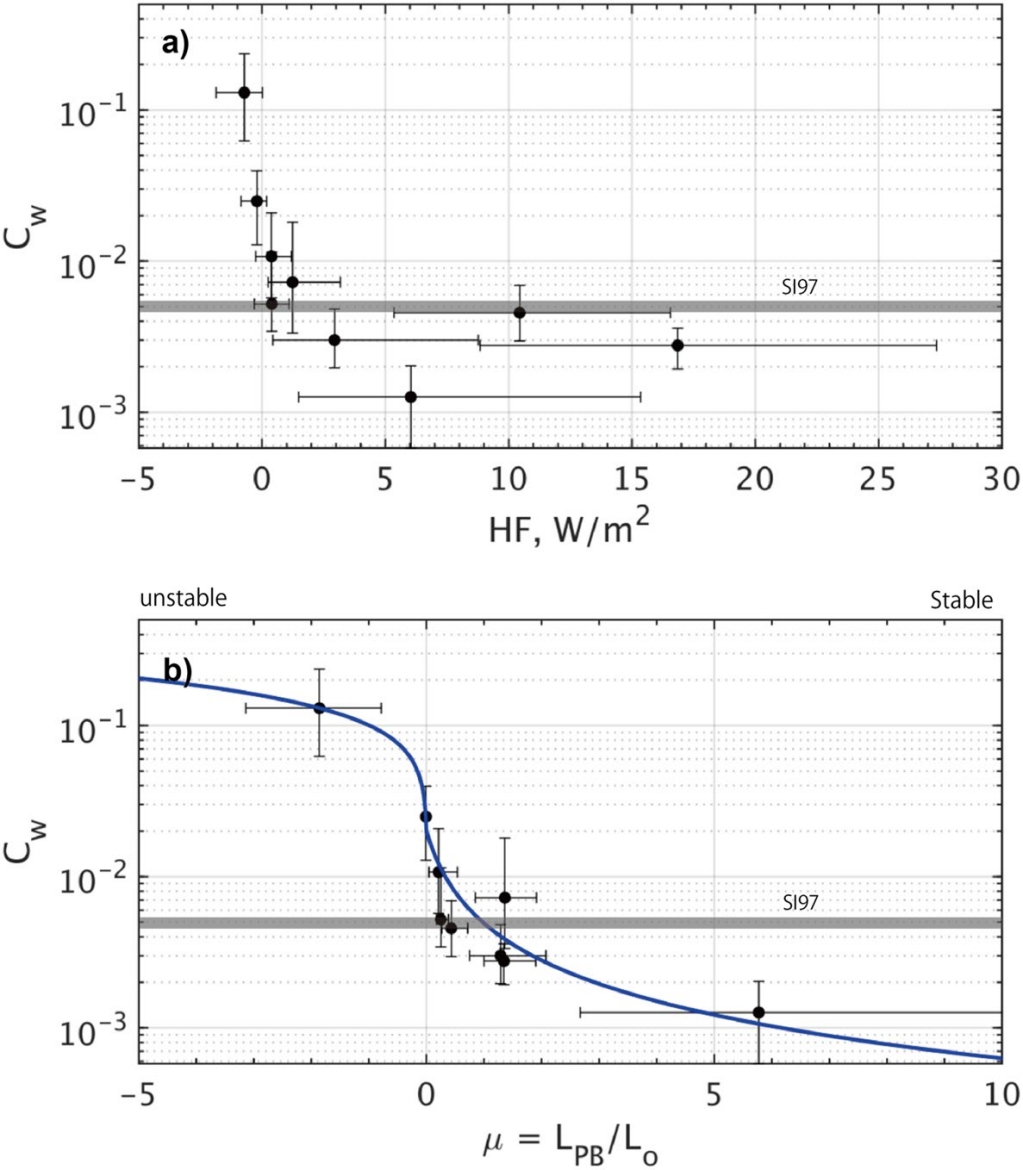
Dependence of $C_{w}$ on (**a**) $F_{h}$ and (**b**) $\mu$, where $\mu = \frac{{L_{PB} }}{{L_{o} }} = \frac{\kappa \langle w'b'\rangle }{{fu_{0}^{*2} }}$. Black dots indicate medians for each station, with first-to-third quartiles shown by horizontal and vertical error bars. Bold blue curve shows the least-square regression deduced from the observed data. Note that the vertical axis is shown on a log scale. Gray horizontal lines represent the constant suggested by Shirasawa and Ingram^30^.

The changing rate of $C_{w}$ as a function of $U_{0}$ exhibits considerable variability among different ice stations (Fig. 3e). For Stations #1 to #5 in mid- to late- August, the data points are distributed to fit the curves of $C_{w}$ = 1 to 5 $\times$ 10^−3^ (Fig. 6a–e). These values for $C_{w}$ appear slightly smaller yet but remain within the same order of magnitude as those derived from some previous studies employing direct observations using the eddy covariance method (c.f., $C_{w}$ = 5.4 $\times$ 10^−3^ by Shirasawa and Ingram, 1997^30^, horizontal line in Fig. 7). In contrast, for Stations #6 to #9, $\tau_{w}$ exhibits a higher rate of increase with $U_{0}$, indicating a significantly larger value of $C_{w}$ ranging approximately from 10 to 100 $\times$ 10^−3^ (Fig. 6f–i).

By considering the median values, the universal characteristics of $C_{w}$ derived from the ECS are extracted in the context of their dependence on the turbulent heat flux ($F_{h}$) and the non-dimensional stability parameter $\mu$ (Fig. 7). The scatter diagram exhibits a pronounced feature of the $C_{w}$ curve, notably decaying with increasing values of $F_{h}$ and $\mu$.

For the analogy of the ice-air boundary layer physics^41^, $C_{w}$ can be expressed as a function of $\mu$ (blue curve in Fig. 7b):

6 $$C_{w} \left( \mu \right) = \frac{\kappa }{{\sqrt {\left( {\ln \left( {\left| {z/z_{0} } \right|} \right) - A\left( \mu \right)} \right)^{2} + B\left( \mu \right)^{2} } }},$$

where $z$ is taken as the planetary boundary layer thickness, i.e., $\left| z \right| = L_{PB}$ and 340 m; the roughness length $\left| {z_{0} } \right|$ is 4 cm. The similarity functions of $A\left( \mu \right)$ and $B\left( \mu \right)$ are defined for the stable ($\mu \ge 0$) and the unstable domains ($\mu \le 0$), respectively by the linear and nonlinear (the reciprocal of cube root) functions in terms of $\mu$^41,42^:

7 $$A\left( \mu \right) = \left\{ {\begin{array}{*{20}c} {10.0 - a_{1} \left( {1 + a_{2} \mu } \right)^{{ - \frac{1}{3}}} \qquad if \quad \mu \le 0, } \\ {10.0 - a_{1} + a_{3} \mu \qquad if \quad \mu \ge 0,} \\ \end{array} } \right.$$

and

8 $$B\left( \mu \right) = \left\{ {\begin{array}{*{20}c} {b_{1} \left( {1 - b_{2} \mu } \right)^{ - 1/3} \qquad if \quad \mu \le 0,} \\ {b_{1} + b_{3} \mu \qquad if \quad \mu \ge 0.} \\ \end{array} } \right.$$

It should be noted that in the regression model (Eq. 6), the curve seamlessly connects the two domains of $\mu < 0$ and $\mu > 0$ through $A$ and $B$ at the point of $\mu = 0$. The constants $(a_{1} ,a_{2} ,a_{3} )$ and $(b_{1} ,b_{2} ,b_{3} )$ are determined by the least-square method by using a MATLAB function *lsqcurvefit*:

9 $$\left( {\begin{array}{*{20}c} {a_{1} } \\ {a_{2} } \\ {a_{3} } \\ \end{array} } \right) = \left( {\begin{array}{*{20}c} {20.995} \\ {75.986} \\ { - 60.026} \\ \end{array} } \right)\;{\text{and}}\;\left( {\begin{array}{*{20}c} {b_{1} } \\ {b_{2} } \\ {b_{3} } \\ \end{array} } \right) = \left( {\begin{array}{*{20}c} { - 0.009} \\ {24.258} \\ {20.996} \\ \end{array} } \right)$$

This interpretation suggests that for the stable condition, i.e., $\mu \ge 0$, the drag coefficient $C_{w}$ should decrease relatively slowly with increasing $\mu$ (Fig. 7b). Regardless, for the unstable condition of $\mu \le 0$, the value of $C_{w}$ significantly grows as $\mu$ decreases across the neutral point of $\mu =$ 0. Our results also suggest that the melting condition strengthens local stratification near the interface, thereby hindering the momentum transfer due to reduced drag efficiency. On the other hand, a transition toward basal ablation disrupts near-interface stratification, promoting vertical momentum transfer due to increased drag efficiency^24^.

Based on their direct measurements of turbulent momentum fluxes ($\tau_{w}$) at approximately 2.5 and 4.5 m below the ice bottom, Cole et al.^12^ reported that $C_{w}$ varied in an approximate range of 1–20 $\times$ 10^−3^ for the weekly median values, with the minimum occurring during the melt season. Qualitatively, the temporal variation suggested by this study is consistent with their results. However, for more quantitative discussions, our estimate ($C_{w}$ >  > 10 $\times$ 10^−3^ at $\mu \le 0$) is quite large, perhaps due to the relatively short observational period, typically lasting for a few days for each station.

**Figure 8:**
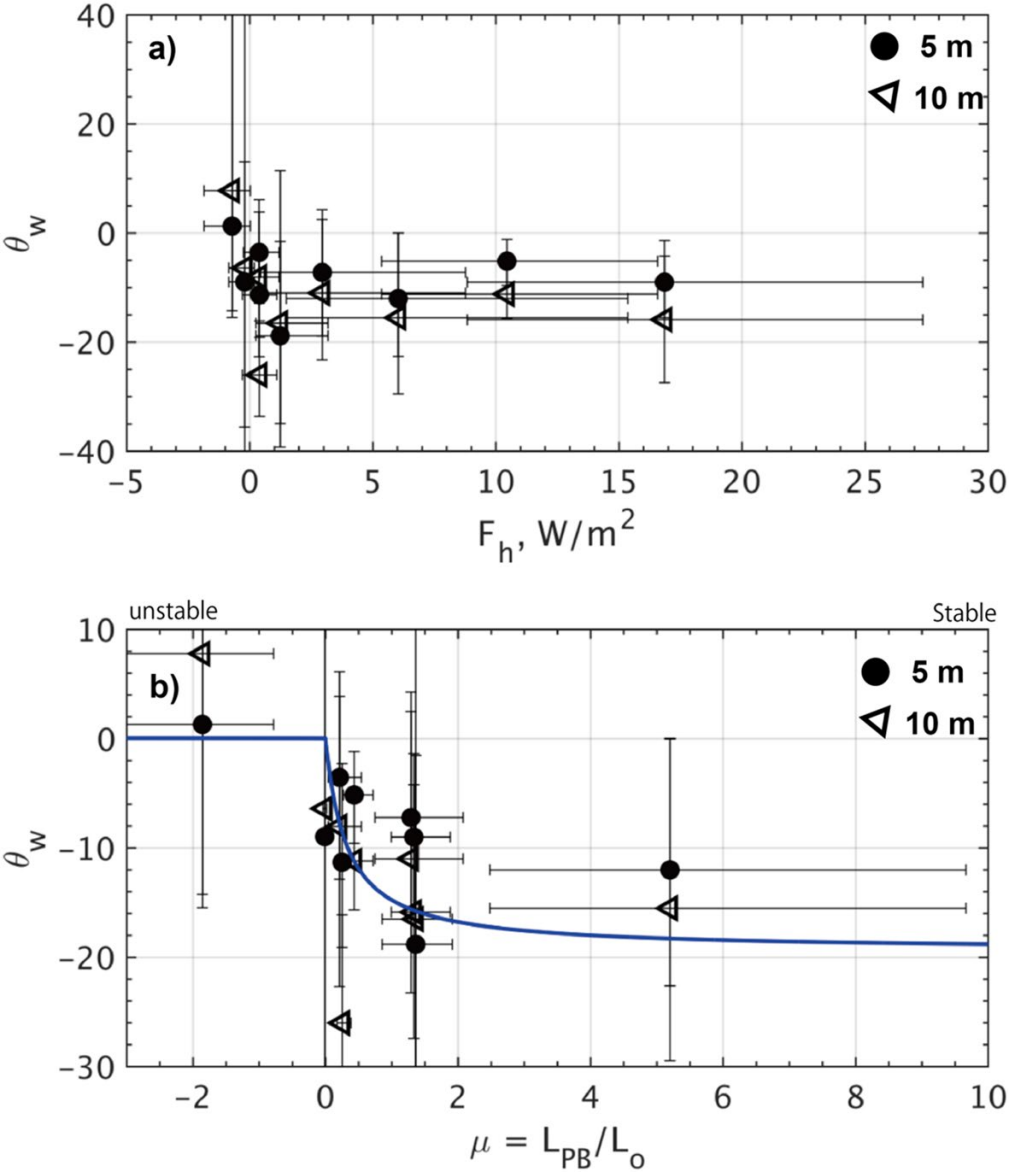
Same as Fig. 7 but for the turning angle $\theta_{w}$. Bold circles and triangles denote the values at 5 and 10 m from the ice bottom. In (**b**), a blue curve shows the solution of $\theta_{w} \left( \mu \right)$ between the near-surface current and the geostrophic current, constructed with the regression coefficients for $C_{w} \left( \mu \right)$.

According to the ADCP observations, for Stations #1 to #8, the turning angle ($\theta_{w}$) exhibited the certain relationship against the parameters of $F_{h}$ and $\mu$. Unfortunately, the statistical dispersion of an interquartile range for $\theta_{w}$ (indicated by error bars) is too wide, typically exceeding 10°, to argue for a universal curve for $\theta_{w}$ as a function of $F_{h}$ and $\mu$ (Fig. 3f; Fig. 8). However, it is evident that the clockwise rotation by $\theta_{w}$ tends to increase with increasing $F_{h}$ and $\mu$ for more stable conditions (Fig. 8). This can physically be interpreted that the Ekman flow rotates more quickly with depth in stable stratification, whereas it rotates more slowly in unstable stratification^10^.

The ice-air boundary layer theory^41^ proposes the solution of turning angle $\theta_{w}$ as a function of $\mu$, based on the similarity functions of $A\left( \mu \right)$ and $B\left( \mu \right)$^42^ (blue curve in Fig. 8b):

10 $$\tan \theta_{w} \left( \mu \right) = - \frac{B\left( \mu \right)}{{\ln \left( {\left| {z/z_{0} } \right|} \right) - A\left( \mu \right)}}.$$

The above semi-empirical model largely aligned with the observed variation of $\theta_{w}$ along the coordinate of $\mu$. The solution also provides diagnostic solutions of $\left| A \right|$= 11.0 and $B$ = 0 for the neutral point ($\mu$ = 0). These show a certain discrepancy from the widely-used approximations of $\left| A \right|$= 2.3 and $\left| B \right|$= 2.1^27^, presumably due to the distortion caused by the extreme values at $\mu$ ≤ 0 observed in our data. The validity of the presented constants will be verified after accumulating knowledge from unstable-time surveys.

### Analogy with atmospheric boundary layer

The dependency of the oceanic drag coefficient and turning angles on the static stability presents a strong similarity with the ones in the atmospheric boundary layer in pack ice^9,40,41^. In the stratified air boundary layer, $C_{a}$ tends to be extremely small, typically *O*(10^−4^), and the turning angle from the geostrophic wind is typically as large as $\theta_{a} =$ 15° counterclockwise in the Northern Hemisphere. Meanwhile, in unstable conditions like in the marginal ice zone^43^, $C_{a}$ increases to be 10 times larger, while the rotation decreases approaching $\theta_{a}$ = 0°.

### Influences on sea ice movement

In the multiyear pack ice during mid-summer, warm water in melt ponds or open water in leads is heated by solar radiation. This process potentially weakens the stratification within the air boundary layer, leading to statically unstable conditions. Conversely, during the same period, the oceanic boundary layer remains stable due to the prevalence of fresh meltwater near the surface. In the disparate situation, the value of $Na = \sqrt {\rho_{a} C_{a} /\rho_{w} C_{w} }$ (see Eq. 2) could grow strongly due to the combination of increasing $C_{a}$ and decreasing $C_{w}$. Indeed, it is acknowledged that the wind factor ($\alpha$) varies seasonally by 50% or more between the summer months (July to October) and the winter months (December to March) in the central Arctic^36^. The seasonal variation in $\alpha$ is often attributed to reduced (increased) internal stress among ice floes during summer (winter)^34,35^. Contrary to the general belief, the findings from the present study suggest that the disparate stability states between the air and oceanic boundaries could partly explain the seasonal changes in $\alpha \approx Na$.

### Implication to ice-ocean coupled simulations

Changes in the parameterization of ice-ocean drag play a significant role in ocean dynamics, affecting processes such as seasonality and trends in ocean surface stress, and the dynamics of the mixed layer^17,18^. Sensitivity studies have demonstrated that different parameterizations introduce significant spatial and temporal differences in the distribution of these quantities over the Arctic Ocean compared to models using constant drag coefficients^14^. Most studies replacing the commonly used constant drag coefficient with more accurate representations of ice-ocean friction have shown improved model results^14,17,18^. Primarily, these papers discuss form factors and various parameterizations of friction between the ocean and ice.

From the perspective noted above, the findings from this study provide an important instance of the potentially variant drag effect from the real field. Our results suggest that numerical simulations with invariant $C_{w}$ could significantly underestimate the kinetic energy exchange between the drifting pack ice and the underlying ocean, especially when the ice is growing. This would lead to potential misrepresentation in the simulated large-scale circulation, the sea ice drift and the near-surface currents. We expect that the incorporation of stability-dependent $C_{w}$ could lead to an improvement in the predicted volume export of sea ice through the Fram Strait, which may partly control the pan-Arctic sea ice extent^4,7^.

Technically, the ice-ocean coupled model can incorporate the empirically derived relation (Eq. 6) to update those values based on predictions of the stability parameter $\mu$ (Eq. 4) from the interfacial buoyancy flux $\left( {w^{\prime } b^{\prime } } \right)$ and friction velocity ($u_{0}^{*}$). An estimate of $w^{\prime } b^{\prime }$ requires solving the heat and salt balance around the ice-ocean interface. The calculation should be based on variables averaged over a period of 1 day or perhaps longer to avoid frequent updates in the calculation.

We also emphasize that the relationship for the stability-dependent $C_{w}$ and $\theta_{w}$ were derived from a limited dataset, and therefore, we recommend that the coefficients of the similarity functions (Eq. 7–9) be updated and evaluated in terms of the simulated ice-drift properties and the eventual sea ice distribution.

## Concluding remarks

In this study, we conducted the direct observations of turbulent fluxes of momentum and heat near the ice-ocean interface as well as vertical profiles of ocean currents and sea ice mass balance in the central part of the Arctic Ocean. The observations were conducted at nine ice stations across the Amundsen and Nansen basins as part of RV *Polarstern* expedition PS138 (ArcWatch 1) in August/September 2023 (Fig. 1).

The eddy-covariance measurements refined the estimate of the drag coefficient ($C_{w}$) at the interface, revealing a greater variability than previously assumed, ranging within $C_{w}$ = 1 to 130 $\times$ 10^−3^ (Fig. 3). Our observations demonstrate a strong dependence of $C_{w}$ on the stability parameter $\mu = \frac{{L_{PB} }}{{L_{O} }}$ (Figs. 6, 7). Specifically, $C_{w}$ exhibits an exponential decay as the flow becomes more stably stratified by basal ice melting, and conversely it increases as flows become neutral or slightly unstable due to ice growth. Additionally, from the ADCP observations, the turning angle ($\theta_{w}$) indicated such spatiotemporal variability as a function of $\mu$, with medians ranging from $\theta_{w}$ =  − 18.8° to 1.3° at 5 m depth for the range of $\mu$ =  − 2 to 5 (Figs. 5–8). Overall, the degree of clockwise rotation ($\theta_{w} < 0$) tends to increase for the stable condition, while it decreases for the neutral or unstable condition.

In the present study, the in-situ data from unstable conditions were limited to the early transitional period during the late summer in the central Arctic, when the cold front of the ice temperature did not reach the ice-ocean interface yet (Fig. S10c-h). Future observations should aim to cover a wider range of variability for $C_{w}$ and $\theta_{w}$ regarding the stability parameter, especially focusing on the negative domain of $\mu$ during the mid-winter.

## Methods

### Study domain

All observations presented here were obtained as part of the research expedition ‘PS138/ArcWatch-1’^37,38^ to the Arctic Ocean onboard the German ice breaker RV *Polarstern* (Alfred-Wegener-Institut;^44^. During this expedition, nine ice stations (Stations #1 to #9) were conducted in the Nansen and Amundsen Basins between 8 August and 19 September 2023 (Fig. 1a).

Station #1 was located at (84.07°N, 31.21°N) northeast of Svalbard, and Stations #2 to #4 were located farther east at the similar latitudes (see Table 1 and Fig. 1a for precise locations). Stations #5–#7 were located on ice floes within the approximate meridional range of 110°–130°E, where Station #7 was positioned near the geographical North Pole. Stations #8 and #9 were positioned along the 60°E line, respectively at latitudes of 88.0°N and 85.5°N.

### Ice thickness variation

Ice thickness observations based on helicopter-borne electromagnetic (EM) induction sounding^45^ were repeatedly conducted during the expedition. The airborne surveys show that the mean ice thickness statistically fell within a range of 1.3–1.4 m for the observational areas (personal communication, J. Rohde). Snow thickness atop the ice was typically around 0.1 m or less.

The ECS instrument was selectively deployed on undeformed part of each ice floe, where the thickness ranged approximately between 1.2 and 1.5 m. It is noted that at Station #1, the ice floe was uniformly thin, measuring approximately 0.75 m throughout.

### Turbulent flux measurements

Measurements of momentum and heat turbulent fluxes were collected using an eddy-covariance technique^46–48^ during all nine ice stations (Fig. 2). The ECS consisted of a 600 MHz Nortek Vector (herein referred to as Vector) three-dimensional current meter (Nortek, Norway) combined with a highly accurate RINKO-EC ARO-EC-CM (herein referred to as RINKO-EC) temperature probe (JFE Advantech Co., Japan). The ECS was deployed through a hydrohole in the ice, and the Vector transducer was positioned approximately 0.5 to 0.8 m beneath the ice bottom. The spearhead of the RINKO-EC sensor was positioned within 10 mm from the lateral boundary of the sampling volume (diameter = 14 mm; height = 14 mm), located about 157 mm from the central transmitter, and inserted at an angle of 45 degrees from below relative to the vertical axis of the water current measurement by the Vector.

The Vector sampling rate was set to 8 Hz during 256-s burst intervals. The ECS collected 2048 data samples per burst, and a burst was set to be recorded every 10 min. The current velocities ($u, v, w$) were measured in the instrument-fixed Cartesian coordinate ($x, y, z$), where a positive $z$ denotes an upward direction. The Vector current velocity is stated to have a nominal precision of ± 1 mm s^−1^ or ± 0.5% for the measured value. The RINKO-EC response time for the temperature is stated to be < 0.3 s^48^. The sensor was calibrated at the manufacturer prior to the expedition, ensuring a precision of ± 0.002 °C.

Prior to the spectral calculations, the raw data underwent conventional postprocessing. Attitude information recorded onboard such as heading, pitch and roll were utilized for the correction of inclination within 5 degrees. Data outside these criteria were discarded. Outliers beyond three standard deviations were removed, followed by interpolation with a linear function^31^. Subsequently, the deviations were extracted by demeaning the sequential records. The cross-power spectral density (CPSD) was calculated using P. Welch’s periodogram method^49^, where 512-sample subsegments were Hanning windowed with a half-overlapping. The vertical fluxes were estimated by integrating the CPSD in the frequency range between $\omega_{1} =$ 0.0039 Hz and $\omega_{2} =$ 1 Hz^50^:

11 $$\langle w^{\prime } c^{\prime } \rangle = \mathop \smallint \limits_{{\omega_{1} }}^{{\omega_{2} }} S_{wC} \left( \omega \right)d\omega ,$$

where $\omega$ represents the frequency, and $S_{wC}$ denotes the CPSD between vertical flow $w$ and an arbitrary variable $C$.

### Vertical profiles of currents

For all ice stations, horizontal ocean current velocities were obtained using a Nortek 1 MHz Signature1000 ADCP^51^ (Fig. 5). The ADCP was deployed through a hydrohole within 5 m from the ECS location as the transducer looks downward. Three-dimensional current velocities were collected at a sampling frequency of 10 min and a burst interval of 1 min, with a sampling rate of 1 s during each burst. The vertical extent of the current vector typically ranges from 1 to 20 m from the ice bottom, with a vertical bin size of 1 m and total of twenty bins. Current velocity was recorded in the instrument-fixed Cartesian coordinate system (XYZ). During post-processing, horizontal velocities with a Percent Good ≤ 60% were discarded. The reported precision of the ADCP is ± 0.3% of the measured current speed.

### Far-field temperature and salinity

Far-field temperature ($$T_{w} )$$ and practical salinity ($$S_{w}$$) are calculated as averages of respective hydrographic parameters over 5–10 m depth using profiles obtained by a tethered, free-falling MSS-90L microstructure profiler (Sea and Sun Technology, Germany;^52^ (red filled circles in Fig. 1b). Typically, 20–40 profiles were obtained during each ice station. The MSS was lowered through a hydrohole in the ice, located ~ 30 m from the ECS, and descending to a maximum depth of 300–400 m. Sensors mounted on the instrument included seawater conductivity (SST small), temperature (PT100) and pressure (PA7-100) (CTD) with a precision of ± 0.002 mS/cm, ± 0.002 °C, and ± 0.1 dbar, respectively. The response time of these sensors is approximately 0.15 s, resulting in a vertical resolution of 0.05–0.10 m for the raw CTD variables. The MSS data are preliminary, with an accuracy of 0.05 in practical salinity determined by comparison to data from more accurate shipboard CTD (not shown). This is sufficient for the analysis in this work. The raw data was processed using the MSP toolbox developed by the Institute of Baltic Research (Warnemünde, Germany;^53^. Further observational protocols and data postprocessing related to the MSS-CTD data are described in more detail in Kawaguchi et al.^54^.

### Ice mass balance observations

Ice interior temperatures ($T_{i} \left( z \right)$) were measured by SIMBA-type ice mass balance buoys equipped with ~ 5 m long thermistor strings (SAMS Enterprise, Scotland;^55^ (Fig. 4;^56,57^. The SIMBAs were deployed in undeformed ice on Stations #1, 4, 5, 6, 7, and 8, at a distance within 100 m from the ECS, where initial thickness of the ice was respectively 0.8, 2.0, 1.3, 1.5, 2.7, and 1.3 m, respectively (Fig. 4b). The SIMBAs measured $T_{i}$ at a vertical resolution of 0.02 m, covering the depth range from the ice surface to 4.8 m. The sampling interval was set to every six hours, and the manufacturer reports an accuracy calibrated to ± 0.125 K. The ice-ocean interface is visually identified from a distinct gap between the two media based on 30-s and 120-s heating mode temperatures of SIMBAs (green lines in Fig. S10;^58^.

### Thermohaline balance at the interface

One of the main aims of this study is to investigate the ice-ocean drag coefficient in terms of the stability in IOBL (blank red circles in Fig. 1b). However, the ECS had no sensor to measure salinity flux, and consequently it did not allow a direct estimate of the buoyancy flux $\left( {\langle w^{\prime } b^{\prime } \rangle } \right)$ or any parameter related to the interfacial stability. Instead, we tried to deduce $\langle w^{\prime } b^{\prime } \rangle$ based on the estimate of turbulent temperature flux $\left( {\langle w^{\prime } T^{\prime } \rangle } \right)$ from the ECS observation. For that purpose, we first used the following approximation for the heat balance^57,58^:

12 $$\langle w^{\prime } T^{\prime } \rangle \approx L_{f} + \dot{q},$$

13 $$L_{f} = - \frac{{\rho_{i} }}{{\rho_{w} }}\frac{{\partial h_{i} }}{\partial t}Q_{L} ,$$

where $L_{f} \left( t \right)$ is the latent heat, $\dot{q}\left( t \right)$ is the conductive heat flux, $t$ is time, $h_{i}$ is ice thickness, $\rho_{i}$ and $\rho_{w}$ are the density of ice and seawater and 910 and 1023 kg m^−3^, respectively; the constant for latent heat ($Q_{L}$) is 276 kJ kg^−1^.

Using the autonomous ice mass balance observations by the SIMBAs^56^, $\dot{q}(t)$ is calculated from the vertical ice temperature gradient near its bottom^59,60^ according to

14 $$\dot{q} = - \frac{{K_{i} \frac{{dT_{i} \left( z \right)}}{dz}}}{{\rho_{w} C_{p} }},$$

where $T_{i} \left( z \right)$ is the SIMBA-based ice temperatures along the $z$-coordinate. We calculate an average value of $\frac{{dT_{i} \left( z \right)}}{dz}$ over a vertical extent of 0.2 m (equivalent to 10 thermistors) above the ice bottom. The thermal conductivity of sea ice ($K_{i}$) is approximated by^61^:

15 $$K_{i} \approx K_{f} + 0.117\frac{{S_{i} }}{{T_{i} \_btm}},$$

where $K_{f}$ is the thermal conductivity for fresh ice (2.04 J m^−1^ K^−1^ s^−1^) and $S_{i}$ is the salinity of sea ice and assumed to be 6.0. In the equation above, the ice temperature ($T_{i} \_btm$) near the bottom is assumed to be the freezing point of seawater ($T_{i} \_btm = T_{f} \approx$ − 1.8 °C). From the SIMBA observations, $\dot{q}$ is typically directed downward and estimated to be − 6.0, − 1.0, − 1.8, − 4.2, 0 and − 4.0 W m^−2^ respectively for Stations #1, 4, 5, 6, 7, and 8 (Table 1); then it is substituted into Eq. (12).

At Stations #2, #3 and #9, there are no ice temperature profiles available. Thus, we estimated $\dot{q}$ according to a linear vertical profile in $T_{i} \left( z \right)$ deducing the temperatures near top and bottom of the ice column. Regarding the near-bottom temperature, it was set ad hoc to the freezing point of $T_{f} \approx$ − 1.8 °C for average seawater [57). At those stations, the air temperatures were deduced from the mean timeseries profile constructed from the remaining SIMBA temperature data (black dashed curve in Fig. 4a). In the calculation, the ice thickness was assumed to be constant at 1.2 m.

The salinity flux $\left( {\langle w^{\prime } S^{\prime } \rangle } \right)$ at the interface is derived from the salt balance, in which $\langle w^{\prime } S^{\prime } \rangle$ is proportional to the melt rate at the ice bottom^27^:

16 $$\langle w^{\prime } S^{\prime } \rangle = \frac{{\rho_{i} }}{{\rho_{w} }}\frac{{\partial h_{i} }}{\partial t}\left( {S_{i} - S_{0} } \right),$$

where $S_{0}$ is salinity at the interface, and it is derived by solving the following quadratic equation^27,54^:

17 $$mS_{0}^{2} + \left( {T_{w} + T_{L} - mS_{i} } \right)S_{0} - T_{w} S_{i} - T_{L} S_{w} ,\;T_{L} = \frac{{\alpha_{s} }}{{\alpha_{h} }}Q_{L} ,$$

where, $\alpha_{h}$ and $\alpha_{s}$ are the extraction coefficients for temperature and salinity, respectively; $m$ is the slope of ‘freezing line’ for the freezing temperature from the UNESCO formula^62^ as a function of salinity:

18 $$T_{0} = T_{f} \left( {S_{0} } \right) \approx - mS_{0} .$$

In the calculation, the certain combination of $\frac{{\alpha_{h} }}{{\alpha_{s} }} = 50$ and $\alpha_{h}$ = 0.0055 are set considering the double-diffusive effects from fresh meltwater accumulated near the ice-ocean interface^27,31,63^.

The buoyancy flux $\langle w'b' \rangle$ is obtained by an equation^27^ below:

19 $$\langle w^{\prime } b^{\prime } \rangle = \frac{g}{\rho }\left( {\beta_{S} \langle w^{\prime } S^{\prime }\rangle - \beta_{T} \langle w^{\prime } T^{\prime }\rangle } \right),$$

where $\beta_{S}$ are the haline contraction and $\beta_{T}$ thermal expansion factors evaluated at $T_{0}$ and $S_{0}$ following the method of McDougall^64^. In Eq. (19), we directly substituted the readings of $\langle w^{\prime } T^{\prime } \rangle$ from the ECS.

### Supplementary Information

Supplementary Figures.

## Data Availability

The field data obtained during the ArcWatch-1 are retrieved from the links below: ECS =  10.17592/001.2024020601. ADCP = 10.17592/001.2024020602. MSS = 10.1594/PANGAEA.967731. SIMBA = 10.1594/PANGAEA.967430. All calculations and plots were done using MATLAB® version 9.14.0 (R2023b). Programming code will be provided upon request.
