# Supplementary material for: Dependency of the drag coefficient on boundary layer stability beneath drifting sea ice in the central Arctic Ocean
Source: Sci Rep. 2024 Jul 4;14:15446. doi: 10.1038/s41598-024-66124-8 (PMC11224423; doi:10.1038/s41598-024-66124-8)

**Supplementary materials**

**Dependency of the drag coefficient on boundary layer stability beneath drifting sea ice in the Central Arctic Ocean**

Yusuke Kawaguchi, Mario Hoppmann, Kunio Shirasawa,

Benjamin Rabe, Ivan Kuznetsov

Fig. S1: Time series of turbulent fluxes from the ECS observations at Station #1. (top) turbulent kinetic energy ($Q$); (middle) friction velocity ($u_{0}^{*}$); (bottom) turbulent heat flux ($F_{h}$). In each panel, red lines show mean current intensity ($U_{0}$) directly measured by ECS. In the bottom panel, magenta bars denote negative $F_{h}$.

Fig. S2: Same as Fig. S1 but for Station #2.

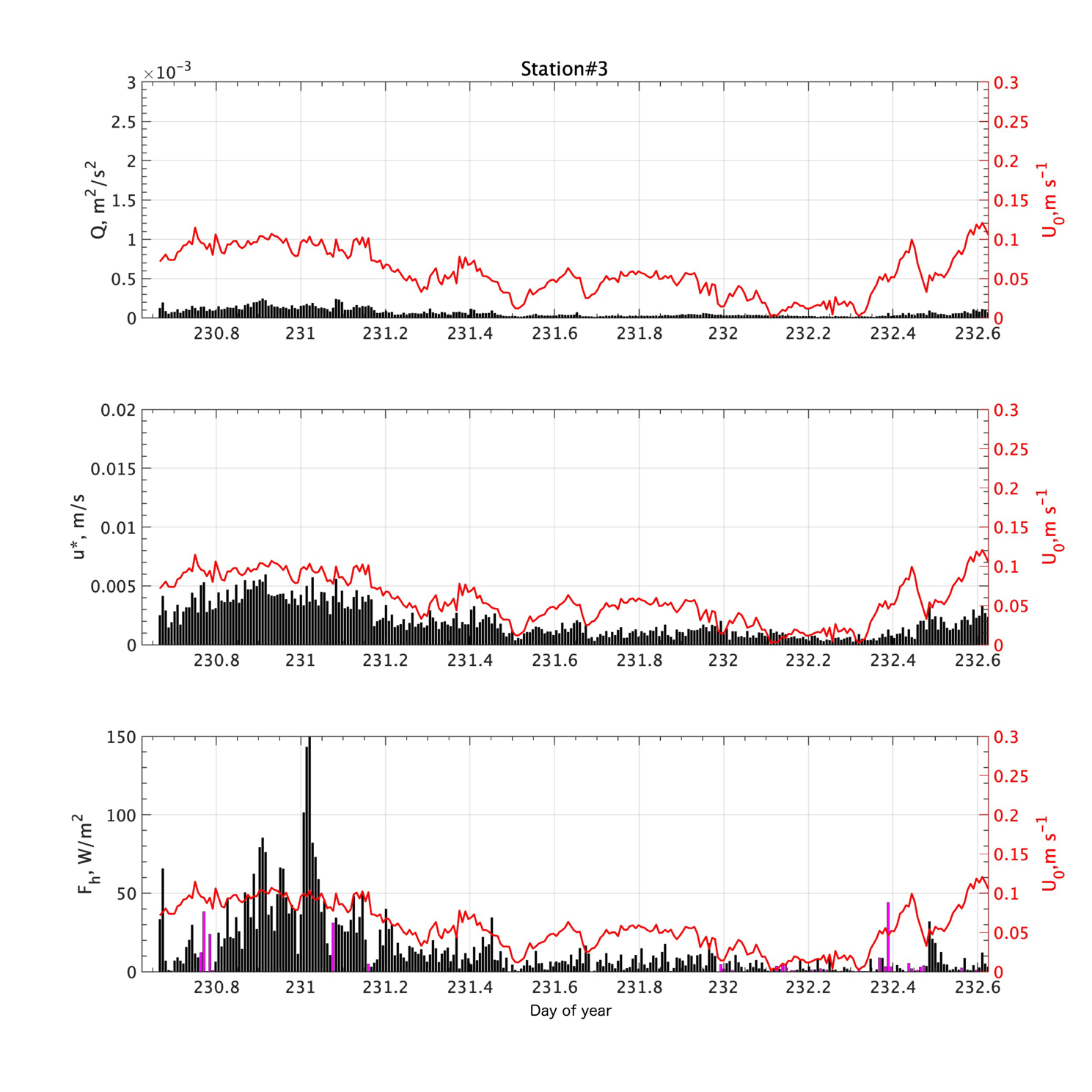
Fig. S3: Same as Fig. S1 but for Station #3.

Fig. S4: Same as Fig. S1 but for Station #4.

Fig. S5: Same as Fig. S1 but for Station #5.

Fig. S6: Same as Fig. S1 but for Station #6.

Fig. S7: Same as Fig. S1 but for Station #7.

Fig. S8: Same as Fig. S1 but for Station #8.

Fig. S9: Same as Fig. S1 but for Station #9.

Fig. S10: Time-vertical sections of relative temperature between ice and water ($T_{i}-T_{w}$) from SIMBA buoys deployed at Stations #1, #4, #5, #6, #7, and #8, respectively for panels (a) to (f). Green lines indicate the ice-ocean interface, detected by the heating mode temperatures.


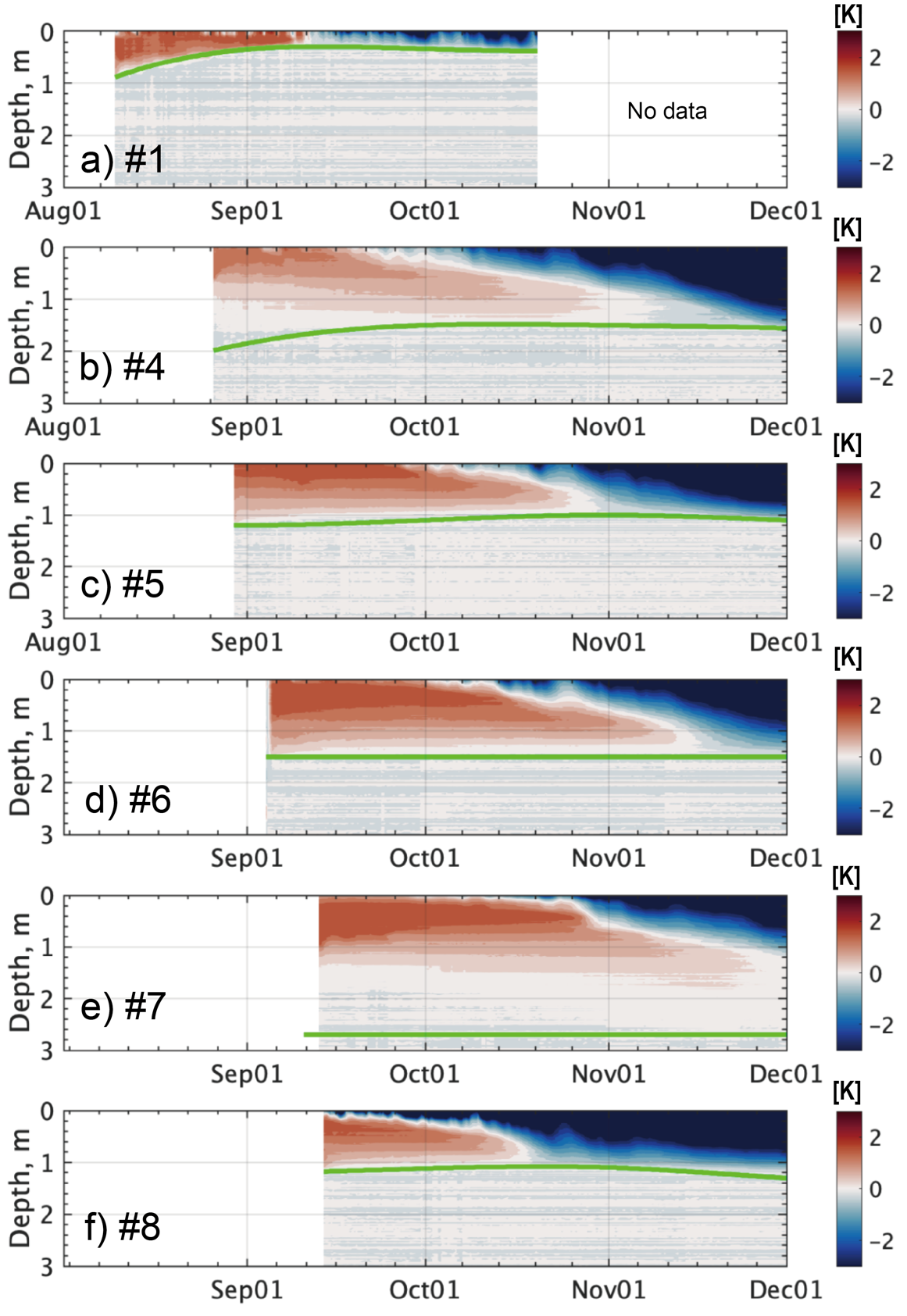

Supplement: Supplementary file 1 — Supplementary Figures. [file 41598_2024_66124_MOESM1_ESM.docx]
